# When Heart Failure Hides a Sarcoma

**DOI:** 10.7759/cureus.95423

**Published:** 2025-10-26

**Authors:** Valter Duarte, Miguel Martins, Daniela Alves, Ana Faustino, Gorete Jesus

**Affiliations:** 1 Internal Medicine, Unidade Local de Saúde da Região de Aveiro, Aveiro, PRT; 2 Cardiology, Unidade Local de Saúde da Região de Aveiro, Aveiro, PRT

**Keywords:** cardiac sarcoma, heart failure, multidisciplinary teams, paliative care, unresectable tumors

## Abstract

Cardiac sarcomas are rare and highly aggressive primary cardiac tumors characterized by rapid growth, local invasion, and early metastasis, most commonly to the lungs, liver, and brain. Clinical presentation is nonspecific and largely depends on tumor location. Diagnosis relies on imaging, with transthoracic echocardiography as the first-line modality, while cardiac magnetic resonance imaging (MRI), computed tomography (CT), and positron emission tomography (PET) provide complementary information for staging, surgical planning, and treatment monitoring. Surgical resection remains the cornerstone of therapy. Chemotherapy, radiotherapy, and emerging targeted or immunotherapies have shown limited benefit, highlighting the need for novel treatment strategies. We describe the case of a 71-year-old woman with multiple comorbidities, including heart failure with preserved ejection fraction, who presented with dyspnea, nocturnal breathlessness, edema, and cough. She was initially treated for decompensated heart failure with good short-term improvement. Echocardiography unexpectedly revealed a 50 × 30 mm left atrial mass infiltrating the mitral valve and septa, producing severe mitral stenosis and regurgitation. Cardiac MRI confirmed a sarcomatous lesion, considered unresectable due to extensive involvement. Staging CT identified an osteolytic lesion in the D12 vertebral body. A biopsy confirmed high-grade sarcoma, and PET showed no further spread. Given the rapid progression and recurrent heart failure, systemic therapy was not initiated, and the patient was transitioned to palliative care, passing away five months after diagnosis. This case highlights the diagnostic and therapeutic challenges of primary cardiac sarcomas. Primary cardiac sarcomas should be considered in patients with unexplained heart failure or intracardiac masses. Multidisciplinary management, improved systemic strategies, and timely palliative care are essential to address the aggressive biology and limited treatment options of this disease.

## Introduction

Primary cardiac tumors are exceedingly rare, with an estimated incidence of approximately 0.02% in autopsy series [1,2]. The majority are benign, including myxomas, lipomas, and papillary fibroelastomas. However, 10-15% are malignant, with cardiac sarcomas representing the predominant subtype [3,4]. Among these, angiosarcomas account for nearly half of all cases, followed by undifferentiated pleomorphic sarcomas, leiomyosarcomas, and intimal sarcomas [4].

Cardiac sarcomas are characterized by rapid growth, aggressive local invasion, and a high propensity for early metastasis, particularly to the lungs, liver, and brain [4]. Cardiac sarcomas may arise in any area of the heart, although angiosarcoma is typically found in the right atrium, whereas fibrosarcoma and undifferentiated sarcoma are found in the left atrium [5]. Clinical manifestations are often nonspecific and largely determined by the tumor’s anatomical location and degree of infiltration of adjacent structures. Left atrial tumors may mimic mitral valve disease or present with systemic embolization, whereas right atrial lesions may cause signs of tricuspid stenosis and pulmonary embolization. Ventricular involvement often results in arrhythmias or conduction abnormalities, while pericardial invasion can lead to pericarditis, pericardial effusion, or cardiac tamponade.

Diagnosis usually begins with a transthoracic echocardiogram, which is the first-line imaging modality for detecting intracardiac masses and assessing hemodynamic impact [1-4]. Cardiac magnetic resonance imaging (MRI) and computed tomography (CT) provide superior tissue characterization, detailed assessment of tumor extension, and surgical planning. Positron emission tomography (PET) is useful for staging and detection of distant metastases, as well as for monitoring treatment response. Although noninvasive imaging is often sufficient to guide management, endomyocardial biopsy may occasionally be required to obtain histological confirmation in cases of diagnostic uncertainty [3].

Surgical resection remains the cornerstone of treatment and is the only intervention associated with improved survival. However, complete resection is rarely feasible and, even in such circumstances, recurrence rates are high and overall prognosis remains poor, with a median survival of six to 12 months [3,6].

Chemotherapy and radiotherapy have been used in neoadjuvant, adjuvant, or palliative settings, but a consistent survival benefit has not been demonstrated [4,7,8]. Doxorubicin monotherapy remains the most commonly employed regimen for unresectable disease, except in leiomyosarcomas, where combination regimens such as doxorubicin with dacarbazine or trabectedin are preferred [4]. More recently, molecular targeted therapies and immunotherapy have emerged as potential treatment strategies [9]. However, robust evidence is still lacking due to the rarity of the disease.

## Case presentation

The authors present the case of a 71-year-old woman with cardiovascular risk factors (non-insulin-treated diabetes mellitus, arterial hypertension, dyslipidemia) under good control, hypertensive heart failure with preserved ejection fraction (HFpEF), hypocoagulated atrial fibrillation, chronic obstructive pulmonary disease, and obstructive sleep apnea syndrome. She presented to the emergency department with dyspnea on minimal exertion, paroxysmal nocturnal dyspnea, peripheral edema, and a productive cough for four days. She denied fever, chest pain, palpitations, syncope, weight loss, night sweats, or other gastrointestinal or genitourinary complaints. Family history was unremarkable.

On admission, she was conscious, afebrile, tachypneic, with bibasilar crackles on pulmonary auscultation and respiratory insufficiency requiring supplemental oxygen. She was hemodynamically stable and presented with bilateral lower limb edema with positive Godet’s sign. Laboratory tests revealed respiratory failure, elevated N-terminal pro-B-type natriuretic peptide of 3023 pg/mL (0-450) and alkaline phosphatase of 123 U/L (46-116), without elevation of inflammatory markers. Chest radiography showed no opacities or pleural effusion. A diagnosis of decompensated HFpEF, possibly triggered by viral respiratory infection, was assumed, and the patient was admitted for stabilization.

**Figure 1:**
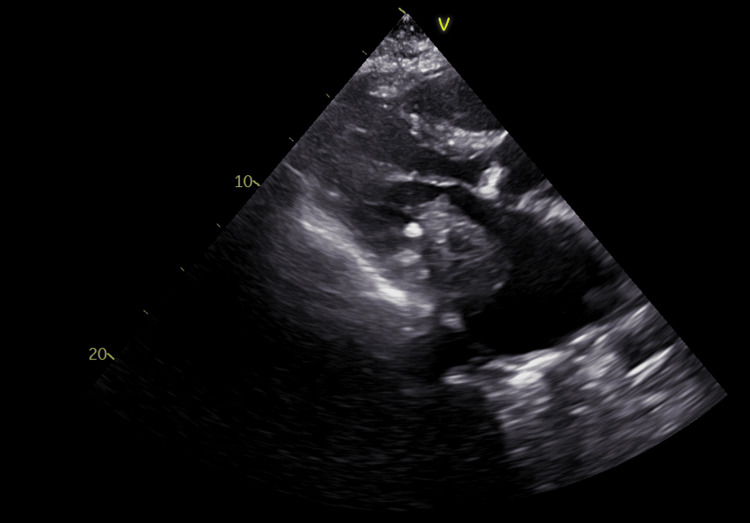
Transthoracic echocardiogram showing the parasternal long-axis view

She showed favorable clinical evolution, with resolution of respiratory failure and peripheral edema under diuretic and bronchodilator therapy. Laboratory workup highlighted iron deficiency without anemia, for which she received ferric carboxymaltose after resolution of the viral infection. A transthoracic echocardiogram (Figure 1) demonstrated a 50 × 30 mm hypoechoic mass in the left atrium (LA), adherent to the mitral valve leaflets along their entire length, infiltrating the interatrial and interventricular septa, causing severe mitral stenosis and regurgitation of difficult quantification. Left ventricular ejection fraction was preserved, the LA was severely dilated, the right ventricle showed moderate systolic dysfunction, and pulmonary hypertension was suspected, with estimated systolic pulmonary artery pressure of 92 mmHg.

**Figure 2:**
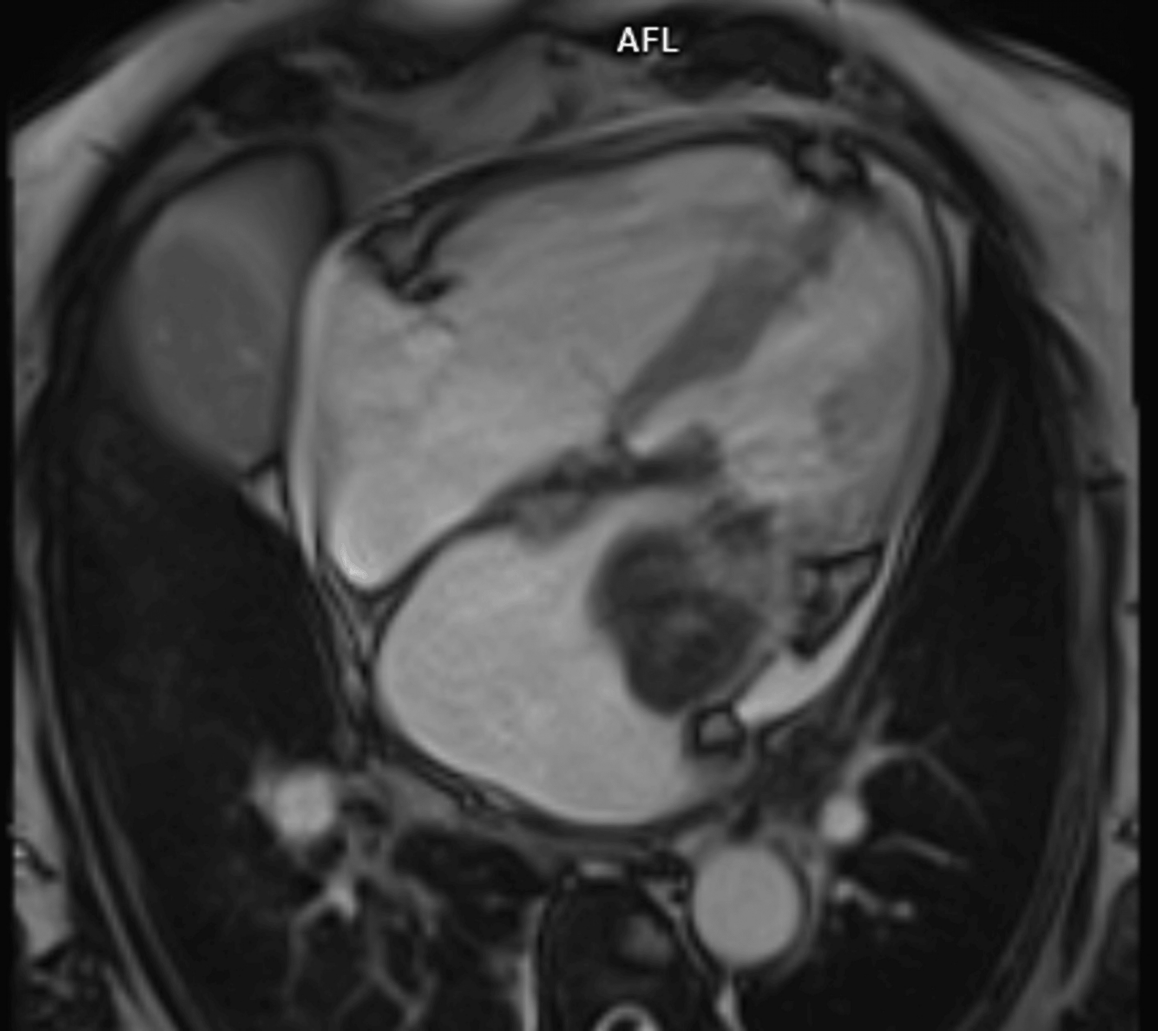
Cardiac magnetic resonance imaging showing the real-time dynamic four-chamber view

Cardiac magnetic resonance imaging (Figure 2) was performed for further characterization and revealed a large, immobile mass with lobulated contours and well-defined borders, attached to the inferior half of the anterolateral, inferolateral, inferior, and septal LA walls and to the posterior mitral leaflet. Contrast uptake was suggestive of a sarcomatous lesion, deemed unresectable after discussion with the cardiothoracic surgery team.

**Figure 3:**
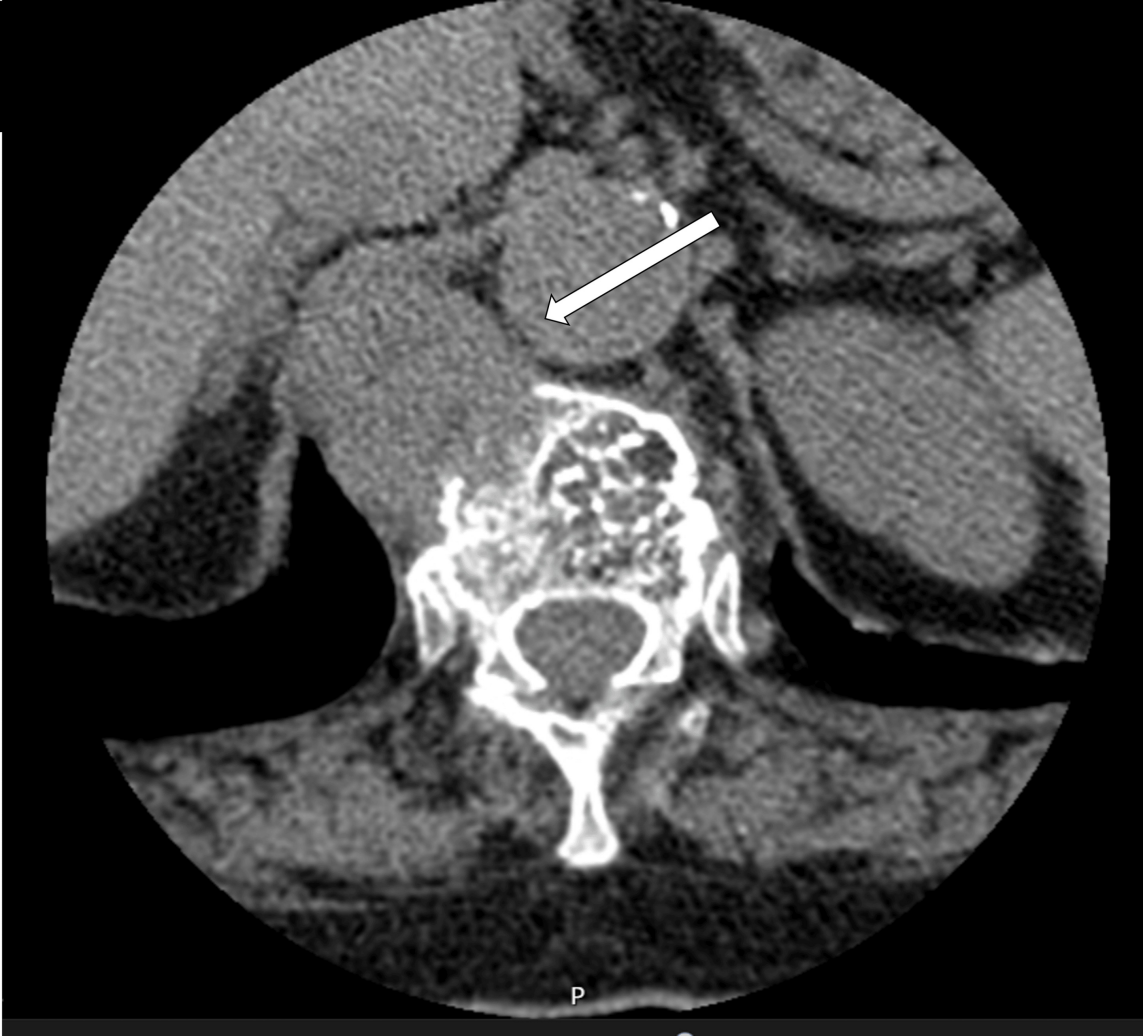
Thoracoabdominopelvic computed tomography scan showing an expansive osteolytic lesion of the D12 vertebral body (white arrow)

A thoraco-abdomino-pelvic CT scan (Figure 3) was requested for staging and revealed a single osteolytic expansive lesion in the D12 vertebral body measuring approximately 37 × 30 mm, suspicious for metastasis. Biopsy confirmed a high-grade sarcoma, and the diagnosis of cardiac sarcoma with bone metastasis was established.

**Figure 4:**
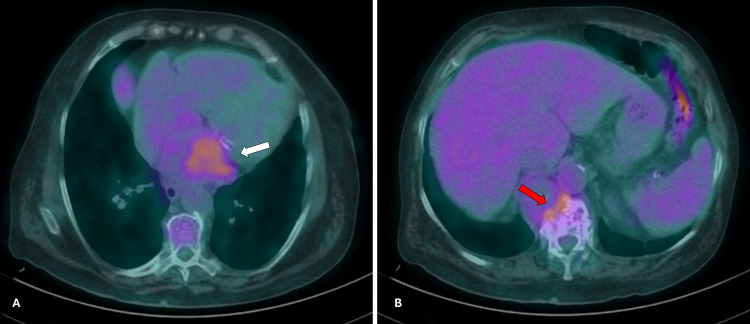
Positron emission tomography showing two hypermetabolic lesions Left side (A) at the level of the left atrium (white arrow). Right side (B) at the level of the D12 vertebral body (red arrow).

She was discharged after 20 days and referred to a specialized sarcoma treatment center. A PET scan (Figure 4) revealed no additional distant lesions. However, due to rapid clinical deterioration with recurrent decompensated heart failure requiring readmission after two months, initiation of directed therapy was withheld, and she was transitioned to exclusive palliative care. The patient passed away five months after diagnosis.

## Discussion

This case illustrates the complexity and diagnostic challenges associated with primary cardiac sarcomas, particularly given their rarity, nonspecific presentation, and aggressive clinical course.

The patient’s symptoms, i.e., dyspnea, edema, and paroxysmal nocturnal dyspnea, were initially attributed to decompensated HFpEF, which was a reasonable working diagnosis given her significant cardiovascular comorbidities. However, further investigation with echocardiography and cardiac MRI revealed a large left atrial mass infiltrating multiple cardiac structures and producing severe mitral stenosis and regurgitation. These findings highlight the importance of maintaining a broad differential diagnosis in patients with unexplained or refractory cardiac symptoms, especially when standard therapy does not yield the expected response.

Despite advances in cardiac imaging, management of cardiac sarcomas remains a significant challenge due to their aggressive biology, late presentation, and limited treatment options. Surgical resection is the mainstay of treatment for primary cardiac sarcomas. Other options as radiation, chemotherapy, and cardiac transplantation, including auto-transplantation, are still controversial due to the rarity of the disease [3]. Despite the overall poor survival rate, patients who do undergo complete resection have better outcomes compared with those who undergo incomplete resection [6-7].

In this case, surgical resection was infeasible because of extensive local invasion. The patient was also found to have early metastatic spread to the vertebral body, further precluding curative intervention. In this context, palliative care plays an essential role in ensuring symptom control and quality of life, underscoring the need for multidisciplinary management and timely referral to specialized sarcoma centers.

## Conclusions

Primary cardiac sarcomas, although exceedingly rare, should be considered in patients presenting with atypical heart failure symptoms or echocardiographic evidence of intracardiac masses. Early and accurate imaging with modalities such as MRI and CT is crucial for characterization, staging, and treatment planning. However, the prognosis remains poor, with high rates of recurrence and metastasis despite aggressive management.

This case emphasizes the need for heightened clinical suspicion, multidisciplinary decision-making, and the development of more effective systemic therapies. Until then, the role of supportive and palliative care remains paramount in managing patients with advanced or unresectable disease, ensuring dignity and quality of life in the face of limited therapeutic options.
